# Three Cases of Pertussis Treated with Bromoform

**Published:** 1893-09

**Authors:** Chas. G. Kerley


### THREE CASES OF PERTUSSIS TREATED WITH BROMOFORM.

Three children, aged respectively eight, six, and four years, members of the same family, developed pertussis within a few days of each other. They came under my observation at the Babies' Hospital at the onset of the disease,—in fact, before the diagnosis was absolutely positive. Pertussis was strongly suspected, however, and they were put on the bromoform treatment at once, which drug has been used in the management of this affection by many observers with widely varying results. It is claimed by some that if bromoform is given early, the disease may be aborted; by others, that the number and severity of the paroxysms will be diminished, and the duration of the attack shortened. Equally good observers, on the other hand, state that after a fair trial the drug proved itself valueless in their hands.

Concerning the cases in question, the youngest, a decidedly rachitic girl of four years, was given five drops four times daily, the other two, fairly healthy boys, each received six drops four times daily. Under the treatment, the disease developed apparently about equally severe in all. The paroxysms varied from fifteen to twenty daily; vomiting occurred frequently during the second week, during which time the disease was most severe, the patients presenting the typical appearance; the eyes congested and the faces puffed and swollen. At about the eighteenth day of treatment, the disease began to subside, the number and severity of the paroxysms diminished rapidly, the vomiting ceased, and at the end of the fourth week, greatly to my surprise, they were practically well as far as the pertussis was concerned.

No other drug was used. Whether the short course and sudden subsidence of the acute symptoms were accidental or due to the treatment, I will endeavor to clear up by further trial.—*Chas. G. Kerley, M. D., in Archives of Pediatrics.*

